# Virtually Being Einstein Results in an Improvement in Cognitive Task Performance and a Decrease in Age Bias

**DOI:** 10.3389/fpsyg.2018.00917

**Published:** 2018-06-11

**Authors:** Domna Banakou, Sameer Kishore, Mel Slater

**Affiliations:** ^1^Event Lab, Department of Clinical Psychology and Psychobiology, University of Barcelona, Barcelona, Spain; ^2^Institute of Neurosciences, University of Barcelona, Barcelona, Spain

**Keywords:** body ownership, embodiment, rubber hand illusion, virtual reality, executive functioning, age bias, implicit association test, Tower of London test

## Abstract

The brain's body representation is amenable to rapid change, even though we tend to think of our bodies as relatively fixed and stable. For example, it has been shown that a life-sized body perceived in virtual reality as substituting the participant's real body, can be felt as if it were their own, and that the body type can induce perceptual, attitudinal and behavioral changes. Here we show that changes can also occur in cognitive processing and specifically, executive functioning. Fifteen male participants were embodied in a virtual body that signifies super-intelligence (Einstein) and 15 in a (Normal) virtual body of similar age to their own. The Einstein body participants performed better on a cognitive task than the Normal body, considering prior cognitive ability (IQ), with the improvement greatest for those with low self-esteem. Einstein embodiment also reduced implicit bias against older people. Hence virtual body ownership may additionally be used to enhance executive functioning.

## Introduction

It has been demonstrated that it is quite straightforward to induce in healthy individuals the perceptual illusion that an object or fake body part is part of their own body—a *body ownership illusion*—illustrating the surprising plasticity of the brain's body representation. For example, the rubber hand illusion (RHI) (Botvinick and Cohen, 1998) has shown that tapping and stroking a rubber hand placed in an anatomically plausible position on a table in front of a person, and synchronously tapping and stroking the corresponding occluded real hand usually leads to the illusion that the rubber hand is their own. This illusion is both subjective and can be measured objectively through “proprioceptive drift”; when asked to blindly point toward their hand, participants will point more toward the rubber than the real hand. Similarly, if the rubber hand is threatened, then there are strong physiological and cortical responses in response to the perceived threat (Armel and Ramachandran, 2003; Zhang and Hommel, 2015). This illusion has been shown to work in immersive virtual reality (VR), where instead of a rubber arm, a virtual arm is seen in stereo 3D as coming out of the participant's real shoulder (Slater et al., 2008). Moreover, a threat to the virtual hand results in a motor response (Kilteni et al., 2012), including motor cortex activation (González-Franco et al., 2013).

Body ownership illusions have also been shown to occur at the whole body level (Petkova and Ehrsson, 2008). In VR a virtual body as seen from first-person perspective (1PP) through a head-tracked head-mounted display (HMD) can be programmed to spatially substitute a person's real body, with motion capture of participants' body movements being mapped to the virtual body in real time. When the person looks down toward their own body, they see the virtual body instead, and when they look toward a virtual mirror, they see a reflection of their virtual body (Slater et al., 2010).

These results demonstrate the high degree of brain plasticity in our body representation, but it is also interesting that the type of virtual body has been found to induce perceptual, attitudinal and behavioral changes in experimental participants, a result first reported in Yee and Bailenson (2007). One characteristic example of this is that when adults are embodied in a small virtual body (van der Hoort et al., 2011) they overestimate the sizes of objects, and when the small virtual body depicts that of a child they also have implicit attitudes and behavioral changes toward becoming child-like (Banakou et al., 2013; Tajadura-Jiménez et al., 2017). However, when they are placed in an adult body that is scaled down to match the size of the child one, then they do not exhibit such changes. In other examples, when White participants experience the RHI over a black rubber hand, or a body ownership illusion over a Black virtual body in VR, this leads to a reduction of their implicit racial bias against Black people (Peck et al., 2013; Farmer et al., 2014; Maister et al., 2015), an effect that has been found to last at least 1 week (Banakou et al., 2016). It was recently shown that when White participants are embodied in a White or Black body, and interact with a Black or White virtual human, the skin color of their virtual body, rather than their real body, influenced which virtual partner they mimicked more (Hasler et al., 2017).

These changes that the body type seems to carry may also apply at higher levels of cognitive processing rather than only at a perceptual and behavioral level. In the study described in Osimo et al. (2015) people were embodied in a virtual body that represented a famous counselor—Dr Sigmund Freud—or alternatively a virtual look-alike representation of themselves. It was found that a strong body ownership illusion when the counsellor's body was Dr. Freud allowed them to find a more satisfactory solution to a personal problem, and positively influenced their mood compared to when the counselor was a double of themselves. Being embodied as Freud had an effect over and above being embodied as a copy of themselves, as if some of the cognitive attributes of a famous therapist mapped over to the participants.

**Figure 1:**
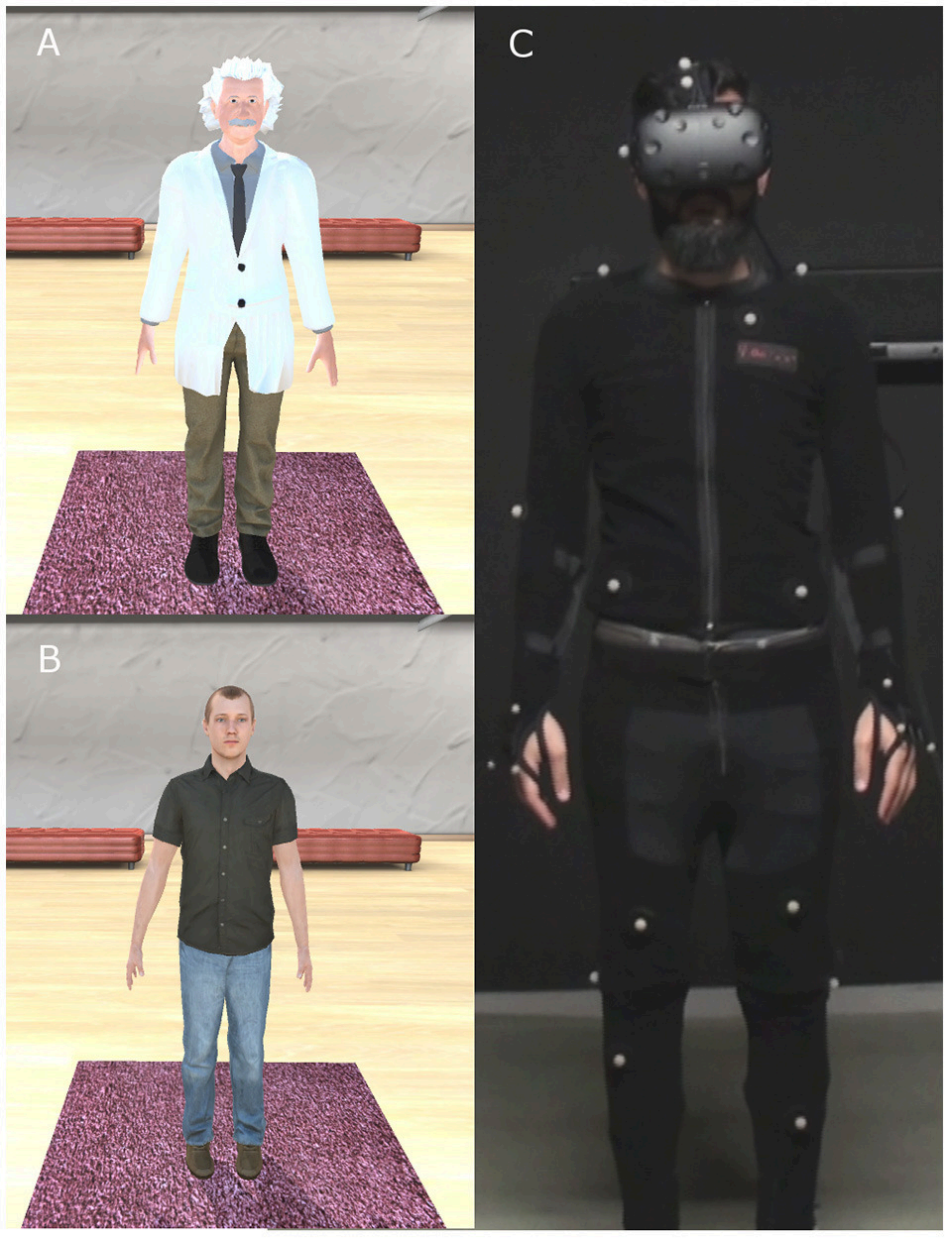
The experimental setup. The body of the participant was substituted by a gender-matched VB, viewed from 1PP, onto which body and head movements were mapped in real time. **(A)** The Einstein virtual body. **(B)** The Normal virtual body. **(C)** Participants were fitted with an HTC VIVE head-mounted display, and their body movements were tracked by 37 OptiTrack markers.

In this paper we investigated whether embodiment of people in a virtual body that is strongly associated with high performing cognitive abilities would result in them exhibiting enhanced cognitive performance. Specifically, we tested whether embodiment in a body that signified super-intelligence, Albert Einstein, would lead to measurable short-term changes in cognitive abilities. In order to accomplish this we used the tower of London task (Shallice, 1982), which was designed to specifically assess executive functioning, and is linked to fluid intelligence and working memory (Unterrainer et al., 2004; Zook et al., 2004; D'Antuono et al., 2016). We were interested to see whether people virtually represented as Einstein would show greater performance on this test, compared to pre-exposure baseline performance. Furthermore, since the virtual body of Einstein was older (Figure 1A) than our experimental group that consisted of young males, we addressed a second issue, that of implicit bias against older people. Specifically, we were interested in examining whether embodiment in an older looking virtual body could lead to a reduction of implicit age-based discrimination in young adults as was found in Oh et al. (2016).

To test this, we ran an experiment with adult males who were embodied in the body of Einstein or, as a control, that of a young adult (Normal). The participants saw their assigned virtual body from 1PP where the eyes of the virtual body coincided with the person's real eyes, and the virtual and real body were spatially coincident. Body ownership over their virtual body was enhanced using the technique of visuomotor synchrony, so that through real-time motion capture, the movements of the participant were mapped to the movements of their virtual body, following earlier examples (Banakou et al., 2013; Banakou and Slater, 2014; Osimo et al., 2015; Tajadura-Jiménez et al., 2017).

## Materials and methods

### Ethics

The experiment was approved by Comissió Bioètica of Universitat de Barcelona. All participants gave their written informed consent prior to participating. The study was performed according to institutional ethics and national standards for the protection of human participants. Ethical considerations included informed consent, right to withdraw, and confidentiality. Exclusion criteria were epilepsy, use of medication, recent consumption of alcohol, intellectual disability and mental health difficulties (e.g., requiring medication). Following completion of the experiment, participants were debriefed with an explanation about the purpose of the study.

### Materials

The experiment was conducted in a Virtual Reality laboratory (width: 3.5 m, length: 4.0 m—back wall to curtain—height: 2.5 m). Participants were fitted with an HTC VIVE head-mounted display (HMD) (Figure 1C). This is stereo and has a nominal field-of-view of 100°, with a resolution of 2,160 × 1,200 pixels per eye displayed at 90 Hz. Participants were also required to wear an OptiTrack full-body motion capture suit that uses 37 markers used with the Motive software to track their body movements in real time (Figure 1C). The infrared technology was implemented with a 12-camera truss by OptiTrack. The virtual environment was implemented on the Unity3D platform. The animation-enabled model of the Normal virtual body was purchased from Rocketbox Libraries and the Einstein model was created with Mixamo Fuse and customized appropriately for the purposes of the study using Mudbox 2016 and Maya 2016 academic versions.

### Participants

Thirty adult male healthy participants aged 18–30 years (28 students and 2 unemployed) (mean ± SD age 22.0 ± 2.81), with correct or corrected vision, were recruited by advertisement and email around the campus of the University of Barcelona. They had no prior knowledge of the experiment, and no or little prior experience of virtual reality. The experimental groups were comparable across a number of variables, including previous experience of VR, and time spent playing computer games (Table 1). Participants were compensated for their participation, by receiving €15 (€5 after the end of the first phase, and the remaining €10 after the end of the second phase).

**Table 1:** Experimental design and distribution of participants by condition.

	**Body**	
	**Normal**	**Einstein**
Male	*n* = 15	*n* = 15
Mean ± SD Age	21.7 ± 3.08	22.2 ± 2.60
Median Code Previous VR Experience (IQR)	4(2)	2(4)
Median Code Games (IQR)	3(3)	2(2)
Mean ± SD fsiq	106.3 ± 7.40	100.5 ± 10.99
Mean ± SD Self-Esteem	34.3 ± 4.22	30.2 ± 4.83

For each case the total number of participants, mean of ages, median and IQR values for participants' experience in VR and hours per week of playing video games (1 = 0, 2 = “<1,” 3 = “1–3,”…,6 = “7–9,” 7 = “>9.” Codes refer to a 1–7 Likert scale. For previous VR experience hours spent playing video games 1 means the least and 7 the most. The Word Accentuation Test (WAT) scores are converted to full scale IQ estimates (*fsiq*), and Self-Esteem scores refer to Rosenberg's scale with higher scores indicating higher self-esteem. Details of these scores are given below.

### Experimental design

The experiment was conducted as a between-groups design with a single factor referred to as “Body,” with levels Einstein (they had the Einstein body) (Figure 1A) or Normal (they had a young male adult body (Figure 1B). The size of the virtual environment and proportions of the content were equivalent to real-life sizes and proportions, and identical in both conditions (Einstein, Normal). Participants were randomly allocated to one of the two conditions. The experimental design can be seen in Table 1. Participants visited the laboratory twice, once to complete some baseline measurements (see Response Variables below), and second a week later for their virtual exposure and the collection of further post-exposure data.

### Procedures

Participants attended the experiment at pre-arranged times. Upon arriving, they were given an information sheet to read, and after they agreed to continue with the experiment, they were given a consent form to sign, and completed a demographics questionnaire. Participants were first assessed with the Word Accentuation Test (WAT) (Del Ser et al., 1997), which is used to estimate intelligence. This test is an adaptation of the North American Adults' Reading Test (NART) (Blair and Spreen, 1989) for Spanish speakers. The WAT utilizes low-frequency Spanish words with all accents removed to make the pronunciation ambiguous, and it has been shown that it gives a reliable estimate correlated with IQ in healthy adults (Gomar et al., 2011). Participants were also assessed on Rosenberg's self-esteem scale (Rosenberg, 1965). The IQ estimates and self-esteem scores for each experimental group can be seen in Table 1.

Participants were then seated in front of a desktop computer and completed an age bias Implicit Association Test (IAT) (Greenwald et al., 1998, 2003), and a Tower of London Task (Shallice, 1982), and the results were recorded (variables: *preIAT, scorepre*). After a period of 1 week they returned for the main experiment.

The VR exposure took place in a laboratory where the position of all participants was controlled through Velcro strips on the floor that were used to mark where they should stand during the experiment. When ready to start, the participants were fitted with a head-mounted display (HMD), and the body-tracking suit (Figure 1C). Initially participants were instructed to turn and move their heads and bodies and walk a maximum two steps away from their starting point to prevent them from hitting the walls due to the restricted laboratory space.

Upon entering the virtual environment, participants found themselves in a virtual room where their body was visually substituted by the life-sized Einstein or a young adult virtual body (Normal), seen from 1PP (Figure 1). Their head and body movements were mapped in real-time to the virtual body. They could see this body both by looking directly toward their real body, and also in a virtual mirror. A series of instructions were then given to them from a pre-recorded audio. First, they were asked to perform a simple set of stretching exercises in order to explore the capabilities and real time motion of the virtual body, including movements of their arms, legs and feet. They were asked to continue performing these exercises by themselves and also look around the virtual room in all directions, where they were asked to state and describe what they saw.

After this 5-min orientation period, participants were instructed through audio that they had to complete a task. They were told that a series of numbers (either positive or negative numbers, fractions, or decimals) would appear around them on the walls or floor and that their task was to locate these numbers and order them in ascending order by selecting them with their hands (for details refer to Movie S1, in Supplementary Material). They were shown 11 number combinations in total (4 different numbers at a time), and the task lasted between 5 and 7 min, depending on how fast they were at selecting the numbers. The reason for choosing this task was to engage participants for the total time required for them to stay in the virtual environment, and to constantly reinforce visuomotor synchrony, since by turning around and pointing they would continually be aware of their virtual body and that its movements were their own.

Finally, the HMD was removed, and all participants completed the age IAT and TOL task again (*postIAT, scorepost*), along with a post-experience questionnaire (Table 2). The whole procedure lasted approximately 35 min. Two experimental operators (one female, one male) were present throughout the whole experiment. Further information is given in Movie S1 (Supplementary Material).

**Table 2:** Questionnaire items.

**Variable name**	**Questionnaire statements**
*vrbody*	I felt that the virtual body I saw when looking down at myself was my own body.
*mirror*	I felt that the virtual body I saw when looking at myself in the mirror was my own body.
*features*	I felt that my virtual body resembled my own (real) body in terms of shape, skin tone, or other visual features.
*twobodies*	I felt as if I had two bodies.
*agency*	I felt that the movements of the virtual body were caused by my own movements.

*All questions were scored on a −3 to +3 scale, where −3 meant least and +3 meant most agreement with the statement*.

### Response variables

#### Implicit association test (IAT)

The IAT (Greenwald et al., 1998) was administered on a desktop screen a week before participants' virtual exposure (*preIAT*), and then immediately after their virtual exposure (*postIAT*). The IAT was completed on the same desktop computer screen both times. The age IAT followed the standard IAT procedure (Nosek et al., 2005) where participants are required to rapidly categorize faces (young or old) and words (positive or negative) into groups. Implicit bias is calculated from the differences in accuracy and speed between categorizations (e.g., young people's faces, positive words and old people's faces, negative words compared to the opposite groups). Higher IAT scores are interpreted as the greater implicit age bias, as this signifies longer reaction times and greater inaccuracies in categorizing old people's faces with positive words, and young faces with negative words. Here the response variable of interest was *dIAT* = *postIAT–preIAT* to examine whether the VR exposure led to any change in bias against old. Positive values indicate greater bias. It has been shown that mean IAT scores tend to show slightly stronger associations corresponding to the pairings of the combined block that is completed first (Nosek et al., 2005). To control for this effect, the order of the combined blocks was counterbalanced between participants as proposed by Nosek et al. (2007). The IAT used was downloaded from the Millisecond Test Library and modified with the Inquisit software by Millisecond.

#### Tower of London task (TOL)

The TOL task is designed to assess executive functioning and specifically, planning and problem solving skills (Shallice, 1982), and its reliability has been shown for test-retest purposes (Köstering et al., 2015). In this test, participants are presented with a model where three beads (red, green, blue) are strategically positioned on three rods of descending heights. They are asked to manipulate the beads from a predetermined starting position on a different set of pegs to match the position of beads in the model. There are 12 different problems of graded difficulty, of 2, 3, 4, and 5-move examples, and only 3 moves are allowed per problem. A problem is classified as correct if the end position is achieved in the minimum number of prescribed moves. The algorithm, based on the procedural details adapted from Krikorian et al. (1994), gives 3 points for a successful solution on the first trial, 2 points on the second, 1 on the third, and 0 points if all trials are failed. The total score is the sum of points on all 12 problems, with a maximum possible score of 36. The TOL was administered on a desktop screen a week before participants' virtual exposure (*scorepre*), and then immediately after their virtual exposure (*scorepost*). It was completed on the same desktop computer screen both times. The response variable of interest was *dscore* = *scorepost*−*scorepre* which showed the degree of improvement (positive values) or decline (negative values) in score after the exposure compared with before. The TOL was downloaded from the Millisecond Test Library and modified with the Inquisit software by Millisecond.

#### Post-experience questionnaire

After each exposure a 5-statement post-questionnaire was administered to assess participants' subjective experience (Table 2). A 7-point scale was used ranging from −3 to +3, with “0” indicating a neutral response on each question (with the scale varying from Strongly Disagree, −3, to Strongly Agree, +3). These questions were related to the strength of body ownership (*vrbody, mirror*) and agency (*agency*) over the virtual body—here we require that the levels of body ownership and agency are the same between the two conditions—while others served as control questions (*features, twobodies*).

### Statistical methods

The major interest is to examine whether there are differences between the Normal and Einstein groups on the two response variables: the IAT for age bias (*dIAT*), and *dscore* (the change in score with respect to the problem solving). These comparisons are premised on there being a strong body ownership illusion.

We adopt a Bayesian approach where we can treat both response variables simultaneously in one model. As can be seen in Table 1 the *selfesteem* variable, which had been elicited prior to the VR exposures, differs between the Experimental and Control groups by chance, and thus must be included as a covariate in the model. Similarly, for *fsiq*.

The overall model is as follows:

$$\begin{array}{l} dscore_{i}\sim \;t\left({df,\eta }_{i},\;\sigma_{score}\right) \end{array}$$

where

η_*i*_ = β_*score*,0_+β_*score*,1_*X*_*i*_+β_*score*,2_*F*_*i*_+β_*score*,3_(*X*_*i*_.*F*_*i*_) +β_*score*,4_*S*_*i*_+β_*score*,5_(*X*_*i*._.*S*_*i*_)

(1) $$\begin{array}{r} diat_{i}\sim \;N\left(\beta_{iat,0}+\beta_{iat,1}X_{i},\;\sigma_{iat}\right)i=1,\ldots ,30 \end{array}$$

*t*(*df*, μ, σ) refers to a Student-t distribution with degrees of freedom *df*, mean (or median) μ and scale paremeter σ. Similarly, *N*(μ, σ) refers to a normal distribution with mean μ and standard deviation σ. Here *X*_*i*_ = 1 if the *i*th individual is in the Einstein group and 0 if in the Normal group, *F*_*i*_ is the *fsiq* score and *S*_*i*_ denotes the *selfesteem* score.

These express a linear model akin to an ANOVA or regression model, which can also be written in statistical model notation as:

dscore = Condition + fsiq + Condition × fsiq + selfesteem + Condition × selfesteem

diat = Condition

where *dscore* has a t-distribution and *diat* is normally distributed with the stated standard deviation. A t-distribution for *dscore* was chosen because inspection of the data suggested a fat tailed distribution. However, this parameterisation also allows for the possibility that *dscore* has a normal distribution, which would occur were *df* about 30 or more.

In a first model fit that we carried out *dIAT* used the same model as *dscore* (i.e., including *fsiq* and *selfesteem*) but there was found to be no relationship between *dIAT* and these variables. Hence these were removed for simplicity.

The prior distributions of the parameters β_∗,*j*_ are conservatively chosen to be Cauchy with median 0 and scale parameter *s* = 10, in order to allow for wide variation. The Cauchy distribution, Student-t with 1 degree of freedom, has infinite mean and variance, and 95% of this distribution is between ±127. The σ_∗_ have the same Cauchy distributions but restricted to the range (0, ∞). Ninety five percent of this distribution lies between 0.4 and 254. The prior distribution of df is also the same Cauchy, but restricted to the range 0–30. Ninety five percent of this distribution is between 0.3 and 27.

The results are not sensitive to changes in prior–for example, if the scale parameter *s* = 20 then the same results are obtained (see Results).

## Results

### Questionnaire responses

**Figure 2:**
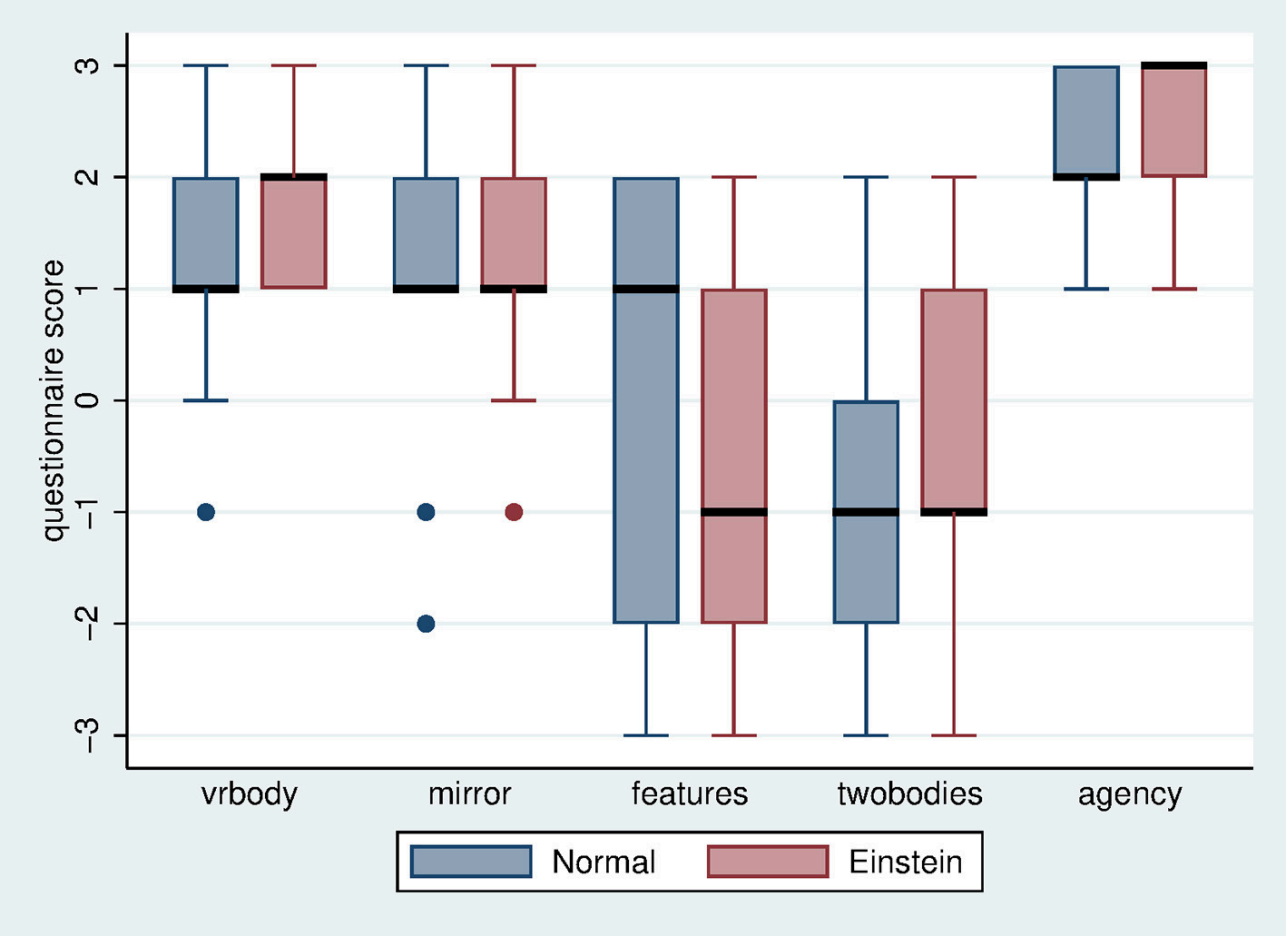
Box plot of questionnaire scores on body ownership and agency. The horizontal thick lines are the medians, the boxes are the Interquartile Ranges (IQR), and the whiskers range from max (min value, lower quartile−1.5^*^IQR) to min (max value, upper quartile + 1.5^*^IQR).

First we consider the responses to the post-experience questionnaire on body ownership and agency (Table 2). The variable *vrbody* refers to the degree to which participants felt as if the body they saw when looking toward themselves was their own body, and *mirror* refers to the body they saw in the mirror. *Agency* refers to the extent to which participants affirmed that the virtual body's movements were their own, whereas the control question *twobodies* refers to the extent to which they felt they had two bodies, and *features* refers to the extent to which participants affirmed that the virtual body had similar physical characteristics to themselves. In Figure 2 it can be seen that the lower quartiles of *vrbody, mirror* and *agency* are all at least 1 in all conditions. The control question *twobodies* always have the upper quartiles at most 1. The score for *features* has the upper quartile at 1 in the case of the Einstein body, but 2 in the case of the Normal body with greater variance. This is not surprising since indeed the Einstein body, being older, had features that would have been most unlike those of the participants. Overall the body ownership and agency scores are very high. This is a pre-requisite for the validity of the study. No further statistical analysis is required here, since we only need to know for this particular sample of people whether or not the body ownership manipulation was successful.

### TOL change

**Figure 3:**
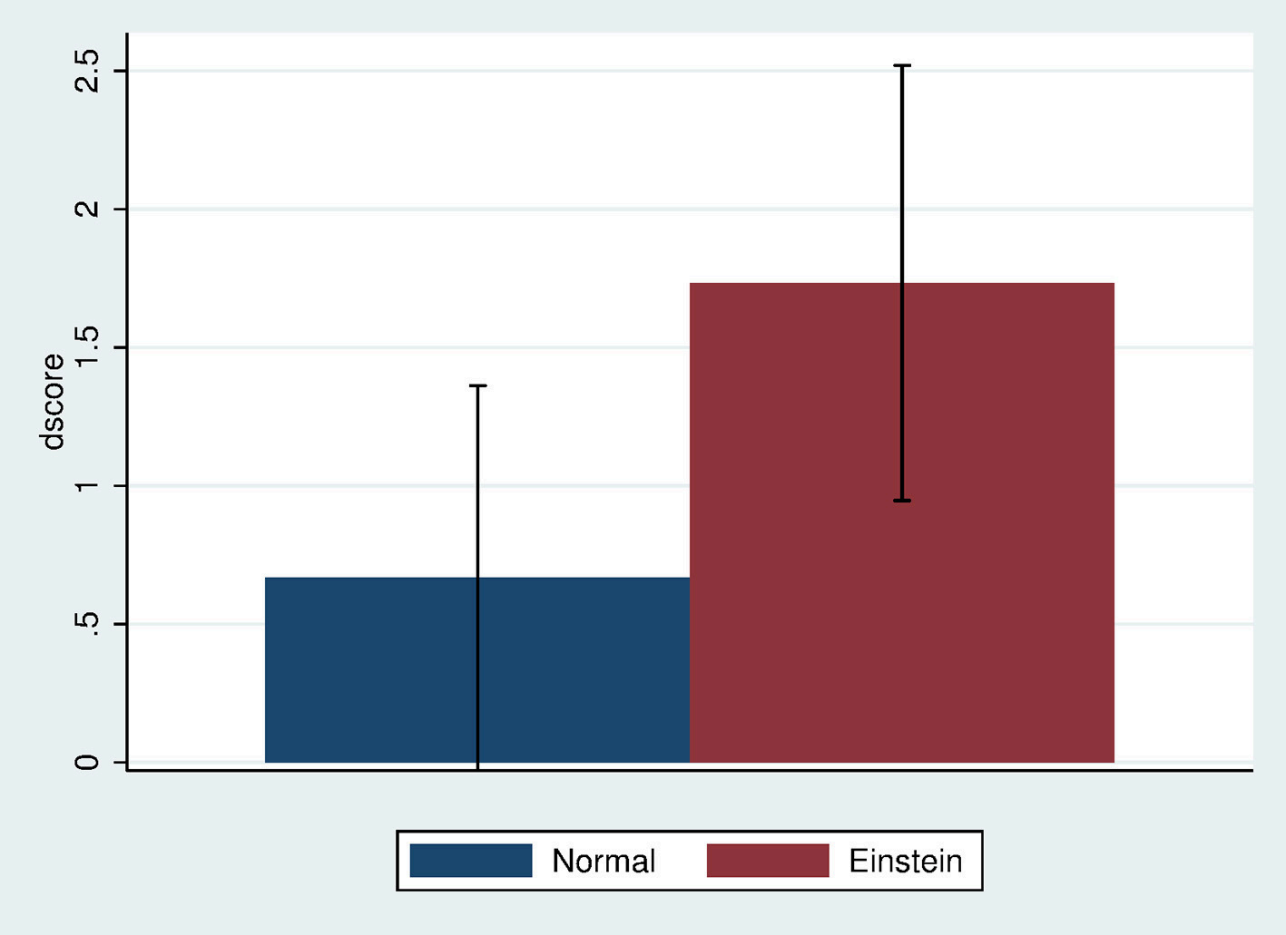
Bar chart showing mean and standard error of *dscore* by Body.

**Figure 4:**
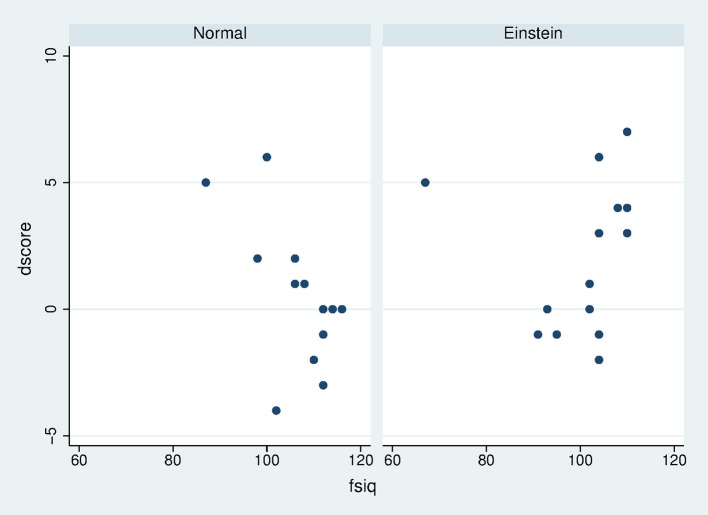
Scatter diagram of *dscore* by *fsiq* and Body.

Figure 3 shows that the mean change in *dscore*, which was calculated as the difference in scores before and after the exposure (*scorepost*–*scorepre*), was greater in the Einstein than in the Normal condition. The means and Standard Errors are 0.67 (0.33 SE) and 1.73 (0.38 SE) with Cohen's *d* = 0.38, which is a small to medium effect size. However, this does not take into account the prior baseline “intelligence” of the participants. Figure 4 shows the scatter diagram of *dscore* on *fsiq*, by condition. Apart from one outlier *dscore* is negatively associated with *fsiq* in the Normal condition while positively associated in the Einstein condition. This outlier was removed for subsequent analysis.

### IAT change

**Figure 5:**
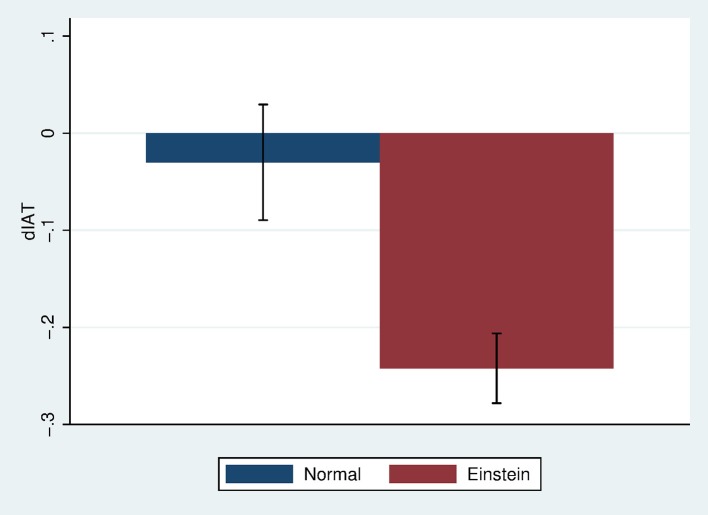
Bar chart showing mean and standard error of *diat* by Body.

**Figure 6:**
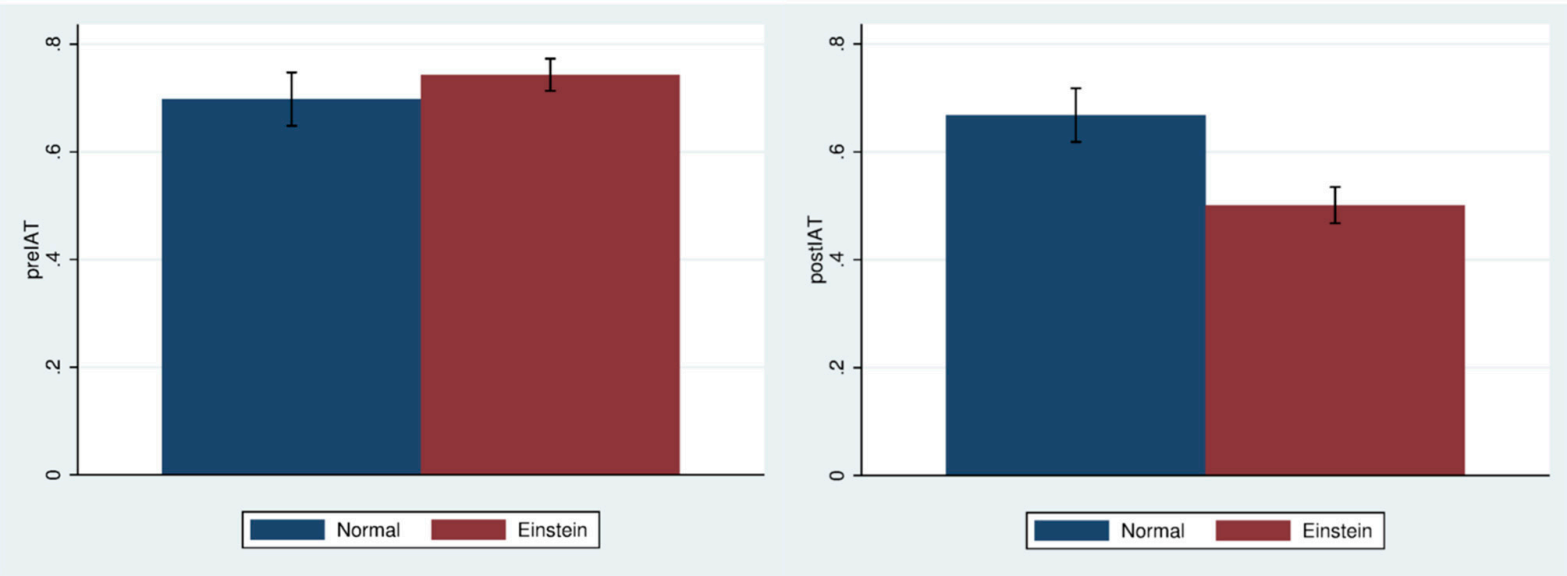
Bar charts showing means and standard errors of preIAT and postIAT by Body.

Figure 5 shows the mean and standard error of the change in IAT by condition. The means and standard errors are −0.03 (SE 0.060) for the Normal body and −0.24 (SE .036) for the Einstein body (Cohen's *d* = 0.54) which is a medium effect size. Figure 6 shows that the bias decreases for those in the Einstein condition, but hardly changes for those in the Normal condition. Hence bias does not change from positive to negative, but rather decreases (in the Einstein condition). This is in line with the findings on racial bias reported in Peck et al. (2013) and Banakou et al. (2016), where we found that embodiment of “White” people in a dark-skinned body reduces but does not flip implicit racial bias.

### Posterior distributions

Table 3 summarizes the posterior distributions. It can be seen from the posterior distribution for *dIAT* that the probability that the Einstein condition results in a smaller *dIAT* is 1−0.068 = 0.932. The posterior probability of interaction effect for *dscore* seen in Figure 4 is 1.000, indicating that for those in the Einstein condition greater *fsiq* scores are associated with a greater problem solving result, whereas the opposite is the case in the Normal condition. However, *selfesteem* has an effect, where greater esteem in the Einstein condition is associated with lesser score (probability = 1–0.008 = 0.992).

**Table 3:** Posterior distributions of the parameters of the model (EQN 1).

**Parameter**	**Coefficient of**	**Mean**	**SE**	**2.5%tile**	**Median**	**97.5%tile**	***P*(>0)**
**dIAT**							
β_*ait,o*_		−0.03	0.001	−0.25	−0.03	0.18	0.397
β_*ait*,1_	Condition	−0.23	0.002	−0.54	−0.23	0.08	0.068
σ_*ait*_		0.41	0.001	0.32	0.41	0.55	1.000
**dscore**							
β_*score,o*_		13.44	0.098	−3.36	13.42	31.10	0.939
β_*score*,1_	Condition	−31.59	0.154	−58.40	−32.02	−4.34	0.008
β_*score*,2_	fsiq	−0.14	0.001	−0.29	−0.15	0.01	0.034
β_*score*,3_	Condition × fsiq	0.44	0.001	0.18	0.44	0.68	1.000
β_*score*,4_	esteem	0.07	0.001	−0.19	0.07	0.34	0.720
β_*score*,5_	Condition × esteem	−0.43	0.002	−0.78	−0.43	−0.09	0.008
σ_*score*_		1.78	0.005	0.86	1.78	2.81	1
*df*		8.35	0.074	1.37	6.12	25.89	1

The first six columns show the means and standard errors, 2.5th, 50th, and 97.5th percentiles of the posterior distributions of the parameters of the model. The seventh column shows the posterior probability of the parameter being positive. The prior 95% credible intervals are −127 to 127 for each of the β_∗_ parameters and 0.4–254 for the σ_∗_. For *df* the prior 95% credible interval is 0.3 to 27.

If instead of using *s* = 10 as the scale parameter for the Cauchy distributions we use *s* = 1, 5 or s = 20, i.e., using priors that are even more conservative, the results hardly change–the equivalents to Table 3 are almost identical.

**Figure 7:**
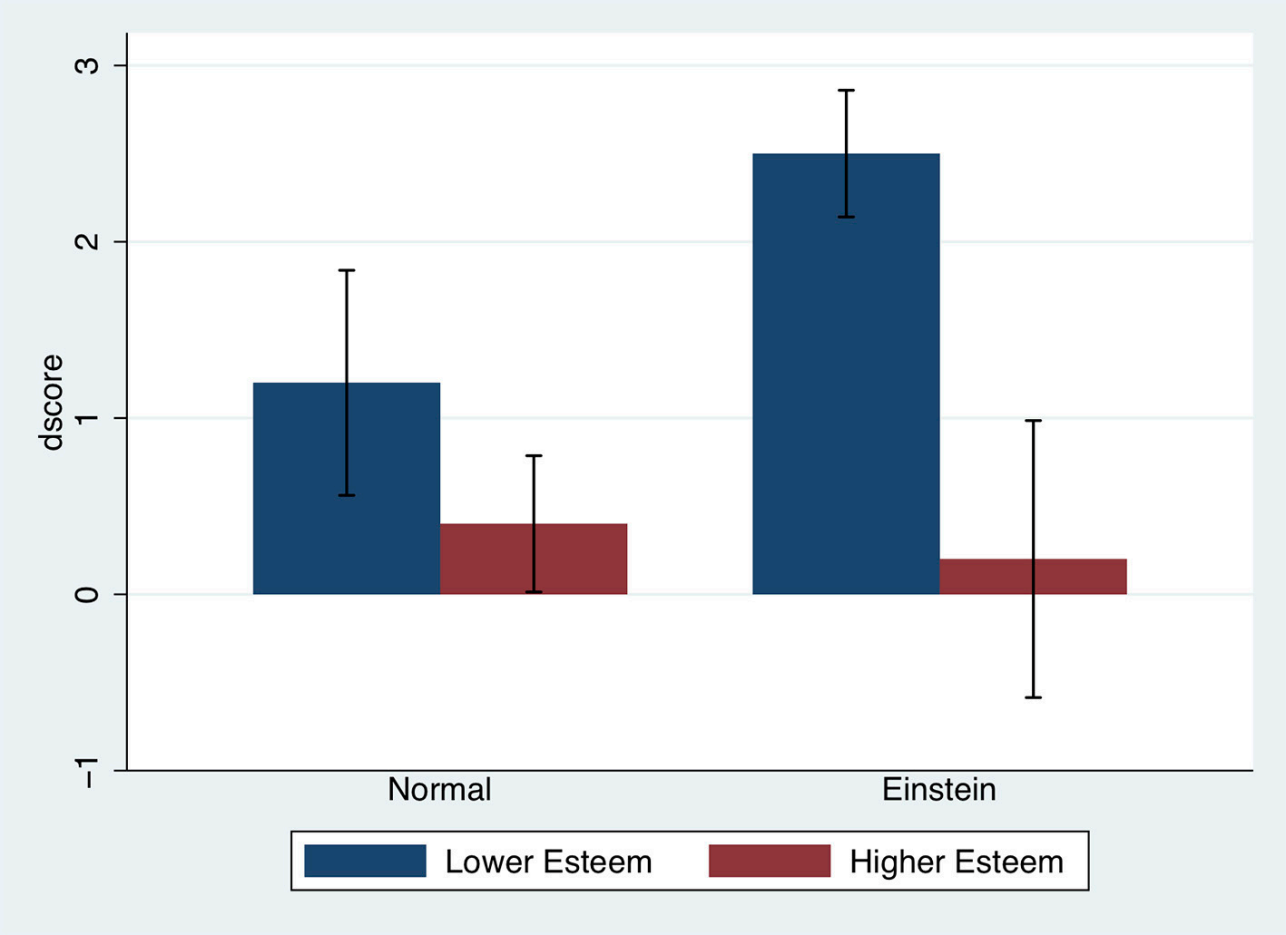
Bar chart showing the means and standard errors of *dscore* by Body and Esteem. Lower Esteem refers to the group with *selfesteem* ≤ median of the sample (32.5) and Higher Esteem to those with *selfesteem* > median.

Figure 7 shows the bar chart of *dscore* by Body and a median split on the *selfesteem* score (median = 32.5). It can be seen that generally those with lower self-esteem had greater improvement in the score compared with those with higher self-esteem. However, the difference between these two is most pronounced in the Einstein condition, and generally the mean change in *dscore* amongst those with high self-esteem is close to zero. This accounts for the apparent negative relationship between *dscore* and esteem for those in the Einstein condition.

### Goodness of fit

Using the posterior distributions of the model we generated 8,000 pseudo random observations on each of the response variables, for each individual–in order to obtain fitted values of ${\hat{diat}}_{i}$ and ${\hat{dscore}}_{i}$ over each individual *i*. The result, referred to as the predicted posterior, is the posterior distribution of each of ${\hat{diat}}_{i}$ and ${\hat{dscore}}_{i}$. We used the mean (over the 8000) as a point estimate of the individual values. These could then be compared with the corresponding originally observed values of the corresponding variables.

**Figure 8:**
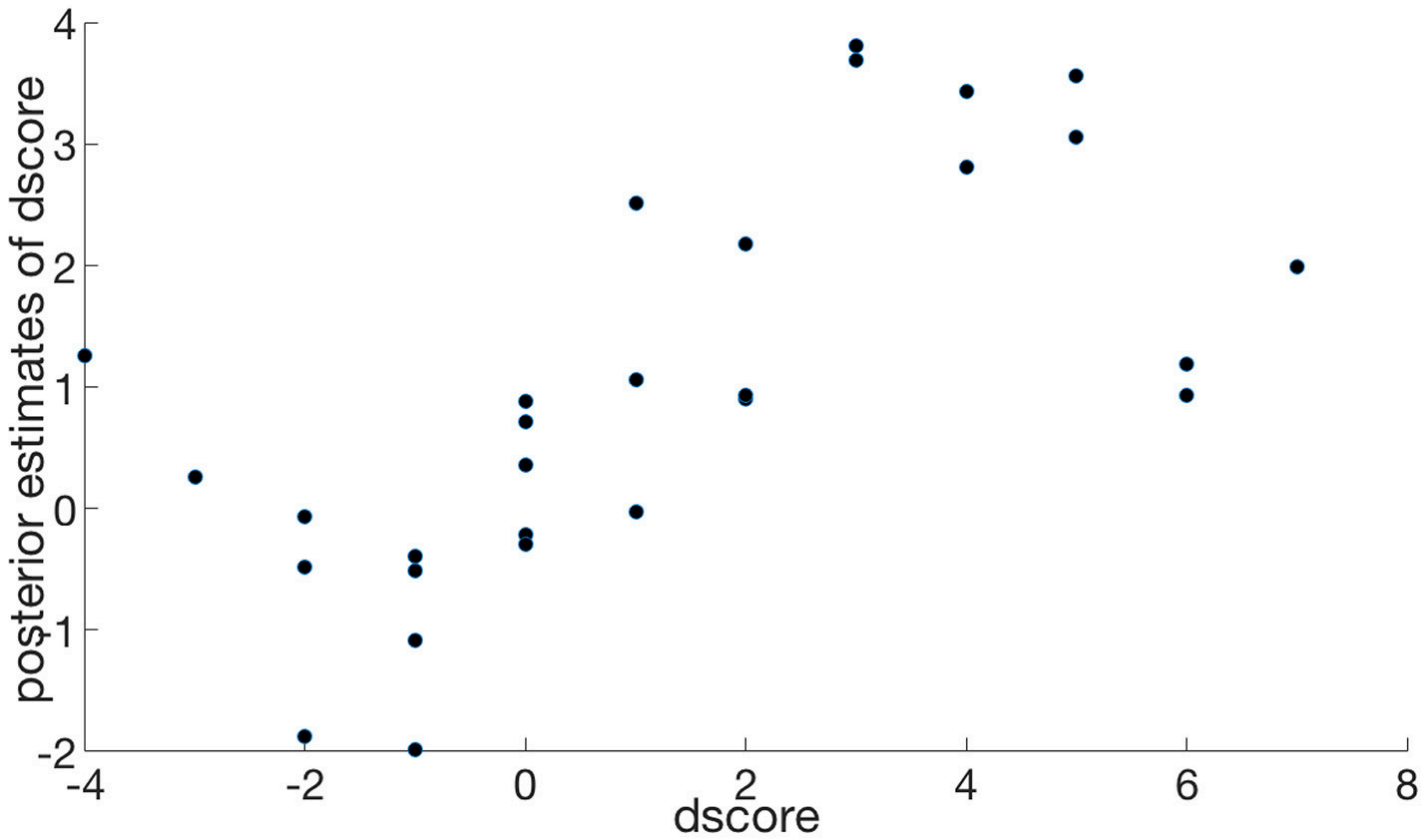
Posterior estimates of *dscore* by observed *dscore, r* = 0.66, *n* = 29.

In the case of IAT the predicted values fall into two clusters, since they are dependent on one binary factor (condition). Therefore, for comparison we compared the means of the observed and fitted values, as shown in Table 4. In the case of *dscore* Figure 8 shows the scatter plot comparing the fitted values ${\hat{dscore}}_{i}$ and the observed values *dscore*_*i*_. In each case the model suggests a good fit to these data.

**Table 4:** Means of the Observed **diat** and Estimated Values $\hat{\text{diat}}\;$from the posterior distribution.

**Condition**	**diat**	**$\hat{\text{diat}}$**
Normal	−0.030	−0.028
Einstein	−0.262	−0.262
Total	−0.142	−0.141

## Discussion

The first result is that although the participants were young men, they clearly had overall a strong illusion of body ownership over a much older body as well as over a body representing one of approximately their own age. Body ownership over bodies profoundly different to the real one has been repeatedly demonstrated. For example, in Slater et al. (2010) the participants were all men, but their virtual body was that of a young girl. In Normand et al. (2011), although participants were thin males, they had the illusion of owning a virtual body with a fat belly. Similarly, Preston and Ehrsson (2016) found that healthy individuals reported illusory ownership over virtual obese or slim bodies during functional magnetic resonance imaging. In Kilteni et al. (2013); Peck et al. (2013); Banakou et al. (2016), and Hasler et al. (2017) all participants were white, but the level of body ownership did not differ among white, black, and even purple-skinned virtual bodies. In the study reported by Banakou et al. (2013), and a recent replication study by Tajadura-Jiménez et al. (2017), it was found that young and older adults felt ownership over a virtual child body or a body of a scaled-down adult, equally and high for both conditions. Osimo et al. (2015) reported a study where young adults experienced ownership over a virtual body that was a 3D scan of their real body and looked very much like themselves, and also a virtual body that was much older and depicted Sigmund Freud, without overall differences in ownership between the conditions.

In line with earlier findings, in the current paper we show that it is possible to induce in young adults a subjective body ownership illusion with respect to a much older virtual body, representing Albert Einstein. Specifically, we show that embodiment induced through 1PP and synchronous visuomotor correlations between the participants' movements and those of their virtual bodies leads to equally high ownership and agency ratings for both those embodied as Albert Einstein and those embodied in a younger looking virtual body. Notably, there was a difference in the subjective report of physical resemblance between participants and their virtual body, which was lower for those in the Einstein condition. As reported in the results, this finding is not surprising since the Einstein body, being older, had features that would have been unlike those of the participants.

Our results also show that embodiment in Einstein leads to changes in implicit attitudes. Specifically, embodiment of young adults in the older Einstein body led to a reduction of implicit bias against elderly, resulting in overall lower IAT scores compared to the control condition (Normal body). Recent evidence suggests that the type of body can indeed have an impact on how the world is perceived and on attitudes and behaviors of the participant (Banakou et al., 2013, 2016; Kilteni et al., 2013; Peck et al., 2013; Maister et al., 2015; Bailey et al., 2016; Tajadura-Jiménez et al., 2017). Regarding stereotyping against the elderly, Yee and Bailenson (2006) used virtual reality to embody participants in a virtual body of a much older person or a body of a young adult. The results showed that negative stereotyping of the elderly was reduced when participants were embodied in the virtual body of old people compared to those embodied in younger.

Our finding expands on these previous findings, demonstrating that the body type carries meaning, and that this meaning has implications for the perceptual processing, attitudes and behaviors of the person experiencing it. This was argued in detail in Banakou et al. (2013, 2016) and Llobera et al. (2013) in the frame of the “cortical body matrix” (Moseley et al., 2012), that not only maintains a multi-sensory representation of the space around the body, but also aspects of the self and corresponding psychological correlates. Moreover, in Banakou et al. (2016) we explained how the IAT is used as a measure of association between categories for any individual, based on statistical associations from the social environment. Similar to there being negative associations with the concept of “Black” people (Greenwald and Krieger, 2006), the elderly also face both implicit and explicit forms of age-based discrimination (Hummert et al., 2002; North and Fiske, 2012; Harwood et al., 2015). Nonetheless, as argued in Maister et al. (2015) and Banakou et al. (2016), during body ownership illusions, the similarity in appearance between the transformed self and the out-group (here Einstein depicting an older person) results in the disruption of associations between the out-group and negative valence items, and substituted by positive associations with the self. Nonetheless, we cannot rule out the possibility that the changes in age-bias scores in our experiment were not caused by the fact that the virtual body depicted only an older person, but by the fact that it depicted a highly eminent universally known person (Einstein). This remains an interesting question to be addressed in future work.

Furthermore, there has been recent evidence that the type of the owned body can result in changes beyond perceptual, attitudinal and behavioral, including also cognitive processing. As introduced earlier, Osimo et al. (2015) used virtual reality to embody people in a virtual body depicting Sigmund Freud. A strong body ownership illusion over that body improved participants' mood and happiness after the experience, and allowed them to find a more satisfactory solution to a personal problem, compared to those who experienced a control body (virtual representation of themselves). The authors explained their findings in terms of activation of perspective-taking mechanisms and the “self” concept. Since the self is associated with attributes of the new transformed body, this allows the participant to access mental resources that are normally inaccessible due to their familiar modes of thinking about themselves. In our case, this generalization of body ownership to higher level capabilities is linked to enhanced performance in cognitive tasks. We show that embodiment in the Einstein virtual body led participants to better performance in a TOL task, which has been linked to fluid intelligence (Unterrainer et al., 2004; D'Antuono et al., 2016). Interestingly, we found that participants' problem solving performance was associated with a measure of IQ and self-esteem scores that differed depending on the embodiment condition (Einstein vs. Normal). We discuss these below and offer possible explanations.

Past studies have shown that most cognitive tasks tend to show improvement with higher IQ (Duncan et al., 1996; Conway et al., 2003; Zook et al., 2004), but also repetition (Strauss et al., 2006; Calamia et al., 2013). However, it has been suggested that performance specifically related to the TOL test is uncorrelated with IQ (Welsh et al., 1991; Bishop et al., 2001; Bechara and Martin, 2004; Huizinga, 2006). Even in samples that were characterized by above-average full-scale IQs, it was found that associations between TOL performance and IQ were not significant, and that even IQs ranging from 80 to 150 were weakly associated with perfect solutions (Luciana et al., 2009). Moreover, Köstering et al. (2015) showed that the TOL can be reliably used for test and re-test in group-based studies and with individual participants.

In our experiment, taking into account the baseline “intelligence” scores of participants, we find that higher IQ is associated with a greater problem-solving result, but only for those embodied as Einstein. However, for those in the Normal condition, IQ and performance are negatively associated, with participants with higher IQ showing weaker results. Therefore, the difference in baseline IQ between experimental and control groups that varied by chance could not have itself accounted for differences between the conditions. But how can it be explained that people with higher IQ in the Normal condition performed worse compared to those in the Einstein condition who performed better?

The authors in Köstering et al. (2015) suggested that the relationship between IQ and cognitive task performance may be strengthened when the task is made more challenging or unpredictable. Similarly, Pekrun et al. (2010) proposed that “boredom” experienced during a task can be expected to reduce both motivation to perform and the effort invested. According to Stankov's hypothesis (Stankov, 1983) individuals with higher scores of intelligence (or higher general ability) might perform worse in simple tasks due to low arousal (boredom), concluding that in such cases intelligence correlates negatively with task performance. We suggest that a similar explanation could apply to our results. Although the task was identical between the experimental and control conditions, the main difference was the type of body participants experienced. Participants in the control condition saw themselves embodied in a young-looking body, with age, and possibly physical characteristics, similar to their own, thus resulting in no additional levels of excitement during the experimental session. This in conjunction with the relative simplicity of the task might have caused a lack of interest, thus driving them to perform poorly. On the contrary, for those participants in the experimental condition there is some new important evidence about the self – “I am Einstein.” This piece of evidence is not a trivial one, it is linked to “super-intelligence.” Seeing oneself as Einstein could have caused participants to reach a higher level of their cognitive abilities (in a way “living up to their name”), thus resulting in better task performance.

The second question that remains is how self-esteem could have affected task performance in the Einstein condition. Concretely, we found that self-esteem scores were negatively associated with task performance for those participants embodied as Einstein. This negative correlation is caused by the change in performance (*dscore*) being high for participants with low self-esteem but with little change in performance by participants with high self-esteem. In other words, there is an increase in TOL score for those with low self-esteem, whereas for those with high self-esteem there is not much change.

Previous research has shown how higher self-esteem is generally associated with higher mental and physical health (Taylor and Brown, 1988; Baumeister et al., 2003; Taylor et al., 2003). Various clinical techniques and standard self-esteem enhancement programs are extensively used to improve self-esteem (Bednar et al., 1989; Frey and Carlock, 1989; Burns, 1993; Mruk, 2006), amongst which are learning techniques of social approval and acceptance (Kernis, 2006), and perspective-taking (Peterson et al., 2015). For example, regarding intimate-partner relationships, it has been shown that low self-esteem participants report increased esteem and closeness toward their partner after going through a traditional perspective-taking technique, whereas participants with more favorable self-views are not affected by the perspective-taking instructions (Peterson et al., 2015). Perspective-taking methods are similar to the technique of embodiment used in our study. The critical difference is that the former is imaginal, whereas virtual embodiment leads to a perceptual illusion of body ownership, without requiring participants to imagine what it would be like to have a different body: they simply experience it. Therefore, as in the above example, it could be argued that giving participants the experience of being Einstein might lead to greatest benefits on cognitive performance for those who have room for improvement–those with low self-esteem. Moreover, since Einstein can generally be considered a socially approved and highly accepted personality, one could argue that this leads to an improvement of self-esteem in low self-esteem people, which is it turn reflected in better cognitive performance.

In line with the above, it has also been suggested self-esteem can be affected by mood, with lower self-esteem people more likely to evaluate themselves positively when they are in a good mood (Brown and Mankowski, 1993). Regarding the underlying process of how moods affect cognition, it has been suggested that self-relevant positive thoughts become more accessible when people are happy, and negative when people are sad (Forgas et al., 1984; Bower, 1987; Brown and Mankowski, 1993). Studies on body representation have shown the impact that one's body can have on emotional state and self-esteem, with participants who feel more positive also showing enhanced self-esteem (Tajadura-Jiménez et al., 2015). In the studies of Osimo et al. (2015) and Tajadura-Jiménez et al. (2017) participants also reported feeling happier after experiencing embodiment in Dr Sigmund Freud or in a child body respectively compared to control groups, however, no data on self-esteem were recorded. Similarly, a potential increase in self-esteem could have affected participants' stress levels as previously demonstrated (Juth et al., 2008). Additionally, stress has been shown to impair cognitive abilities, including selective attention, working memory, and other verbal or visual solving problems (Keinan et al., 1987; Braunstein-Bercovitz et al., 2001; Luethi, 2008; Tiferet-Dweck et al., 2016). There could therefore be the possibility that embodying the Einstein body led low self-esteem participants to increase their self-confidence - thus decreasing any experienced task-related stress - which in turn led to better performance.

Although we cannot draw any conclusions on improvement of self-esteem, motivation, mood, or stress levels based on our data, our speculation is that our findings can be associated with experiencing enhanced self-reassurance, provided that the critical role of the body is taken into account. This is to an extent supported by our findings, as there were changes in cognitive performance only for those people in the experimental condition, and only for those with low self-esteem. Since, as argued earlier, in that condition the “self” is now associated with Einstein, this gives participants access to their own internal mental resources that they might associate with that body that would otherwise be inaccessible. In our case this is specifically linked to enhanced cognitive performance as measured by the TOL task. On these lines, it is currently unclear whether such changes are the product of virtual embodiment in a personality known for intelligence, or the effect is due to an increase in self-esteem, arousal, motivation or mood from virtually embodying any famous universally respected character. Also, we cannot conclude that similar changes would take place were different cognitive abilities to be tested. It remains unclear whether embodiment as Einstein has a specific effect on cognitive processing related only to problem-solving, or the effect can carry over to different cognitive or other functions. Hence additional tasks to control and account for these effects should be further tested. Certainly we do not claim that embodiment in a different body, no matter how prestigious and important personality this body represents, could give people access to entirely new knowledge (e.g., quantum mechanics, physics). However, it could make them more open to acquire such new knowledge.

Additionally, further research is required to understand the contributions of body ownership and agency to these effects. For example, previous research has shown that experiencing agency over the virtual body's movements is an essential factor for the illusion to result in behavioral, perceptual, and implicit attitudes (Banakou et al., 2013; Osimo et al., 2015; Banakou and Slater, 2017). The significance of agency was explicitly addressed in Banakou and Slater (2017), where we found that body ownership in itself cannot account for behavioral after-effects (illusory agency over speaking), and that it is necessary that body ownership be primarily induced by visuomotor synchrony between movements of the participants and movements of the virtual body (always in the context of 1PP over the virtual body). In this work we did not study how asynchronous visuomotor correlations, leading to a reduction of body ownership, might have influenced the results, however, in future studies we aim to replicate and extend these types of findings, and specifically address the agency factor. Although additional research is needed in this direction and to understand the extent to which body ownership can generalize to higher level capabilities, this method could prove useful in the improvement of cognitive performance, especially in people who have low self-esteem.

## Conclusions

The results of this experiment, in conjunction with earlier work discussed above, shows that virtual embodiment can be used to generate an illusion of body ownership of a virtual body that substitutes their own body, through first-person perspective and visuomotor correlations over real and virtual body movements. The main focus here is that embodiment does not only lead to perceptual, attitudinal and behavioral correlates as previously shown, but can also cause changes in cognitive processing. Specifically, our findings suggest that embodiment in a virtual body that is associated with high cognitive abilities, such as Albert Einstein, results in better performance in a TOL task, and also a reduction in age-based discrimination of young adults toward the elderly. There is evidence that participants' baseline “intelligence” and self-esteem correlate with the above findings, taking however into account the critical role of the body in which embodiment occurs. Nonetheless, the present study comes with a number of limitations and alternative hypotheses in the interpretation of the results that we discuss above, which point out the necessity for further research to be able to understand the exact mechanisms resulting in such effects.

## Author contributions

MS designed the original concept. All authors designed the experiment. DB and SK implemented the scenario and carried out the experiment. MS carried out the analysis. DB and MS wrote the paper.

### Conflict of interest statement

The authors declare that the research was conducted in the absence of any commercial or financial relationships that could be construed as a potential conflict of interest.
